# Supervised prediction of drug-induced nephrotoxicity based on interleukin-6 and -8 expression levels

**DOI:** 10.1186/1471-2105-15-S16-S16

**Published:** 2014-12-08

**Authors:** Ran Su, Yao Li, Daniele Zink, Lit-Hsin Loo

**Affiliations:** 1 Bioinformatics Institute, Agency for Science, Technology and Research, 30 Biopolis Street, #07-01 Matrix, Singapore 138671, Singapore; 2 Institute of Bioengineering and Nanotechnology, Agency for Science, Technology and Research, 31 Biopolis Way, The Nanos, #04-01 Singapore 138669, Singapore; 3 Department of Pharmacology, Yong Loo Lin School of Medicine, National University of Singapore, 10 Medical Drive, Singapore 117597, Singapore

**Keywords:** nephrotoxicity, supervised classification, drug toxicity, kidney, random forest

## Abstract

**Background:**

Drug-induced nephrotoxicity causes acute kidney injury and chronic kidney diseases, and is a major reason for late-stage failures in the clinical trials of new drugs. Therefore, early, pre-clinical prediction of nephrotoxicity could help to prioritize drug candidates for further evaluations, and increase the success rates of clinical trials. Recently, an *in vitro *model for predicting renal-proximal-tubular-cell (PTC) toxicity based on the expression levels of two inflammatory markers, interleukin (IL)-6 and -8, has been described. However, this and other existing models usually use linear and manually determined thresholds to predict nephrotoxicity. Automated machine learning algorithms may improve these models, and produce more accurate and unbiased predictions.

**Results:**

Here, we report a systematic comparison of the performances of four supervised classifiers, namely random forest, support vector machine, *k*-nearest-neighbor and naive Bayes classifiers, in predicting PTC toxicity based on IL-6 and -8 expression levels. Using a dataset of human primary PTCs treated with 41 well-characterized compounds that are toxic or not toxic to PTC, we found that random forest classifiers have the highest cross-validated classification performance (mean balanced accuracy = 87.8%, sensitivity = 89.4%, and specificity = 85.9%). Furthermore, we also found that IL-8 is more predictive than IL-6, but a combination of both markers gives higher classification accuracy. Finally, we also show that random forest classifiers trained automatically on the whole dataset have higher mean balanced accuracy than a previous threshold-based classifier constructed for the same dataset (99.3% vs. 80.7%).

**Conclusions:**

Our results suggest that a random forest classifier can be used to automatically predict drug-induced PTC toxicity based on the expression levels of IL-6 and -8.

## Background

The kidney plays an important role in the maintenance of water and electrolyte balance, and the filtration and elimination of metabolic wastes and drugs from the plasma [1]. Due to drug exposure and active transport and metabolism of drugs, the kidney is susceptible to drug-induced toxicity [2-5]. Nephrotoxic drugs may perturb renal perfusion, induce loss of filtration capacity, and cause damage to the vascular, tubular, glomerular and interstitial cells in the kidney [6]. Drug-induced nephrotoxicity can lead to acute kidney injury, or chronic kidney disease that may process to end-stage kidney disease [6-8]. However, nephrotoxicity of drug candidates is often detected only during the late phases of drug development, and accounts for 19% of drug attrition in phase 3 of clinical trials [9]. Therefore, early, pre-clinical prediction of nephrotoxicity could help to prioritize drug candidates for further evaluations, increase the success rates of clinical trials, and reduce the overall time and cost of drug development.

Renal proximal tubular cells (PTCs) are a major target for drug-induced toxicity because they are involved in the regulation of filtrate concentration and drug transportation and metabolism [1]. Current pre-clinical, *in vitro *nephrotoxicity predictors are usually based on protein- or gene-expression markers of immortalized renal proximal tubular cell lines [2,10-13]. Most of these predictors have only been tested in around ten or less compounds [2]. Recently, Li et al. have developed an *in vitro *predictor based on the expression levels of two inflammatory markers, interleukin (IL)-6 and -8, in human primary renal proximal tubular cells (HPTCs) [14] and human embryonic stem cell-derived HPTC-like cells [15]. These markers were tested in a larger number of 41 compounds, and gave higher prediction accuracy than many previous predictors [2]. However, most of these existing predictors use simple linear thresholds to distinguish between the effects of nephrotoxic and non-nephrotoxic compounds, even though more than one markers (or "features") are measured from the cells. These manually-determined thresholds may be subject to human biases, and have difficulties in distinguishing features that are non-linearly separable [16]. Therefore, we wonder if non-linear decision boundaries identified automatically using supervised classifiers can further improve the accuracy of nephrotoxicity predictors based on the IL-6 and -8 markers.

Supervised classifier is a computational algorithm that maps, or classifies, input data into different pre-defined categories based on a set of training data whose category membership is known. The support vector machine (SVM) algorithm is one of the most commonly used classifiers. It constructs classification boundaries based on soft margins that allow mis-classified data points, and is especially useful when the data is not linearly separable and/or the number of features is high [17]. The *k*-nearest-neighbor (*k*-NN) and naive Bayes classifiers are two other commonly used classifiers. In a *k*-NN classifier, the category membership of a data point is determined by a majority vote of its neighboring training data points [18]. Naive Bayes classifier is based on the Bayes' theorem and assumes that the measured features are independent [19]. These two classifiers have the advantages of being simple and efficient, especially for low numbers of features [20,21]. Finally, the random forest algorithm is a relatively new type of classifier based on ensemble learning of a set of decision trees [22]. In certain datasets, random forest may achieve higher classification accuracy than SVM [23]. Despite the popularity of these classifiers, their performances have not been systematically compared and studied under the context of nephrotoxicity prediction.

Here, we report a systematic comparison of random forest, SVM, *k*-NN, and naive Bayes classifiers in predicting the PTC toxicity of 41 well-characterized compounds that are toxic or not toxic to PTC. The prediction is based on the IL-6 and -8 expression levels measured by Li et al. in HPTCs [14]. We describe how the parameters of all the tested classifiers can be automatically determined without any manual intervention. We also compare the importance of IL-6 and -8 in predicting PTC toxicity. Finally, we also show that supervised prediction based on the best classifier, random forest, achieves higher accuracy than the threshold-based classifier used by Li et al. [14].

## Methods

### Dataset

**Figure 1:**
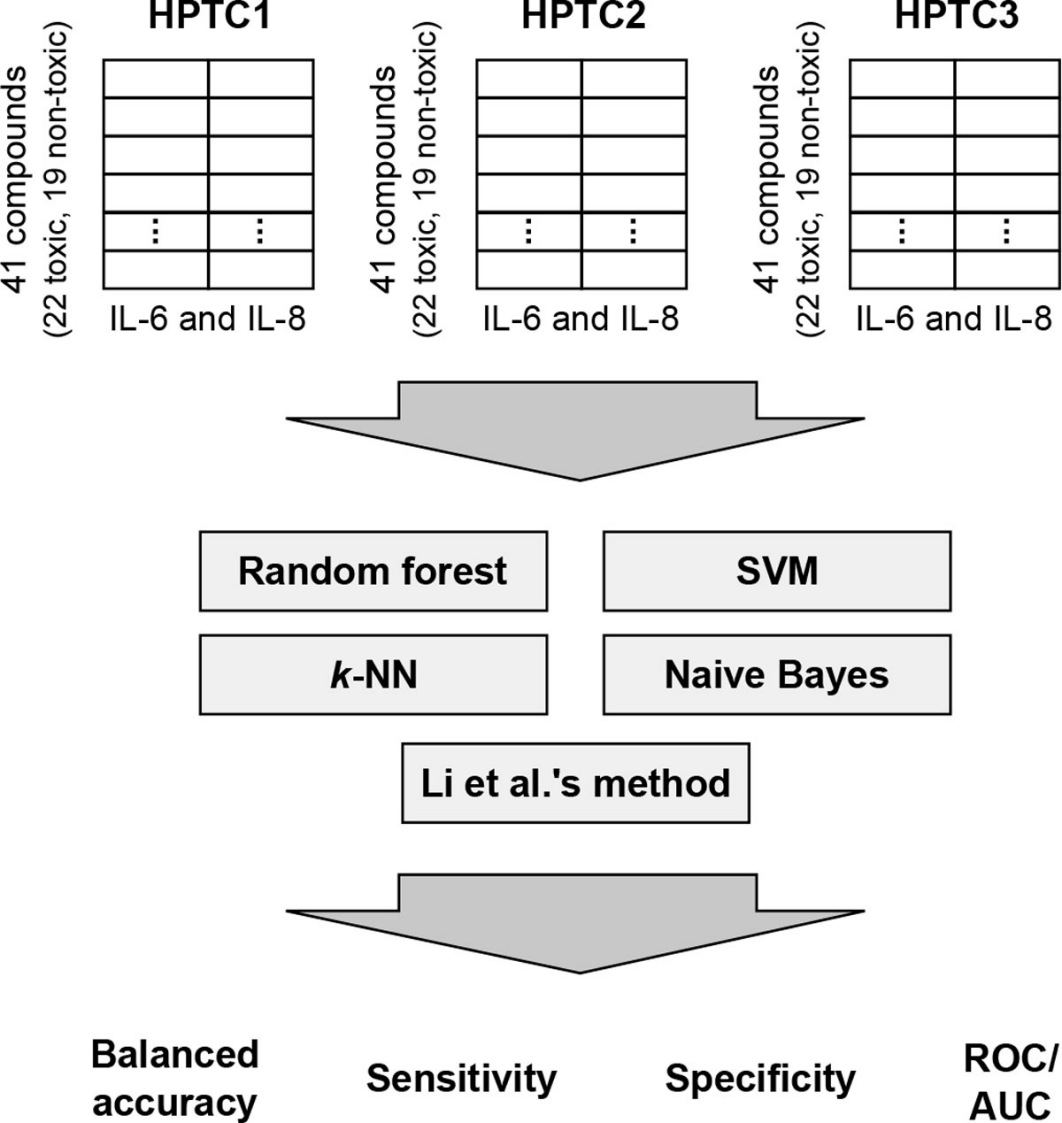
**Overview of our methodology**.

We used the gene expression dataset generated by Li et al. [14]. The dataset was collected from HPTCs derived from three different human donors (HPTC1, 2, and 3). The cells were exposed to 41 compounds for 16 hours, and the expression levels of IL-6 and -8 were determined using quantitative polymerase chain reaction (qPCR). The measured values were then averaged across three experimental replicates and divided by the vehicle control values (Additional File 1). For each batch of HPTCs, we obtained a final 41x2 floating point data matrix and presented it to each of the tested classifiers as described below (Figure 1). IL-6 and -8 were selected because their expression levels are substantial increased in injured or diseased kidneys [24-27]. The 41 compounds can be divided into two categories [2,14]. The 'toxic' category has 22 nephrotoxicants that are known to be directly toxic to the human PTCs. The 'non-toxic' category has 11 nephrotoxicants that are not known to directly damage PTCs, and 8 non-nephrotoxic compounds. A more detailed description of the experimental protocols and compounds can be found in the report of the original study [14] and another more recent study [15]. In these previous studies, simple classifiers based on manually determined thresholds were used, and cross validation was not used to test the performance of these classifiers. The mean balanced accuracy of these classifiers constructed using all the data points (compounds) was reported to be 80.7% [14].

### Classifier evaluation

We compared four different supervised classifiers, namely random forest, SVM, *k*-NN and naive Bayes (Figure 1). The maximum expression levels of IL-8 and -6 induced by the tested compounds were used as inputs to the classifiers. For each classifier, we used a stratified 3-fold cross validation procedure [28] to estimate its generalized classification performance. This procedure randomly divided the dataset into three roughly equal folds, one of which was used to test a classifier trained on the remaining folds. We repeated the whole cross validation procedure 10 times, each with a different random fold division. The final classification performance was obtained by taking the mean of all the obtained measurements.

We used three different classification performance indicators: sensitivity, specificity, and balanced accuracy. The definitions of these three measurements are as the following:

(1) $$\text{sensitivity}=\frac{TP}{TP+FN}\times 100\%,$$

(2) $$\text{specificity}=\frac{TN}{TN+FP}\times 100\%,\;\text{and}$$

(3) $$\text{balanced accuracy}=\frac{\text{sensitivity}+\text{specificity}}{2}.$$

where TP is the number of true positives, TN is the number of true negatives, FP is the number of false positives and FN is the number of false negatives. We performed all the analyses using the R statistical environment (v3.0.2) on a personal computer equipped with an Intel Core i7-3770K processor and Windows 7 operating system. The R source code used can be found in Additional File 2.

### Random forest

Random forest is an ensemble learning method that constructs a large number of decision trees (*B*) during training, and predicts the category label of a new data sample by taking the mode of the labels predicted by these trees [22]. During training, a random subset of *m_rf _*features is selected, and the best spit of data points based on these features are used to construct a decision tree. We tested *B *= 50, 100, 150, 200, 250, 300, 400, 500, 600, 700, 800, 900, 1000, 1200, 1400, 1600, 1800, and 2000. Since our dataset only has two features, we set *m_rf _*= 1. The "randomForest" library (v4.6-10) under the R environment was used to perform random forest classification.

### Binary support vector machine

SVM aims to construct a decision hyperplane with the largest margin that distinguishes data points from different categories [29]. Let us denote the training data and category labels to be $\text{\{}x_{i}\text{,}y_{i}\text{\}}$, where $x_{i}\;\in R^{n}$, $y_{i}\in \left\{-1,1\right\}$, and i=1,…,m. If the data points are linearly separable, the optimum decision hyperplane is w⋅x+b=0, where  is a weight vector that is normal to the hyperplane, and *b *is a bias term. For all the input data , they must satisfy the following constraints:

(4) $$w\cdot x_{i}+b\geq +1\text{ for }y_{i}=+1,$$

(5) $$w\cdot x_{i}+b\leq -1\text{ for }y_{i}=-1.$$

These two equations can be combined as:

(6) $$y_{i}\left(w\cdot x_{i}+b\right)-1\geq 0\forall i.$$

The hyperplane with the maximum margin can be calculated through solving the following quadratic programming problem:

(7) $$\begin{array}{c} \text{mi}{\text{n}}_{w,b}\frac{1}{2}{∥w∥}^{2},\\ \text{subject to }y_{\text{i}}\left(w\cdot x_{i}+b\right)-1\geq 0\;\forall i\text{.} \end{array}$$

To handle non-separable datasets, these constraints are relaxed with a positive slack variable $\xi_{i}$, where i=1,2,...,m (called "soft margin") [17]. Then the optimization in **Equation 7 **becomes:

(8) minw,b12w2+C ∑i=1mξi,subject to yi(xi⋅w+b)≥1-ξ i∀i,ξi≥0.

The upper bound for the error in the training dataset is provided by $C\overset{m}{\underset{i=1}{\sum }}\xi_{i}$, where *C *is a regularization parameter. This optimization equation allows a trade-off between large margin and small error values.

In the case where a decision function is not linear, the data is mapped into a higher-dimensional space, and a hyperplane is constructed so that the data can be linearly separated in this new space. The projection $x^{\prime }=\Phi \left(x\right)$ is done through $\Phi :R^{N}\to F$. This hyperplane that separates the two categories has a similar form as the linear case: $w\cdot x^{\prime }+b=0$. The optimization equation is also similar to **Equation 8 **except that the input now becomes$x^{\prime }$. A Lagrangian is constructed and can be transformed into a dual form:

(9) $$\begin{array}{c} \text{max }L_{D}=\underset{i}{\sum }\alpha_{i}-\frac{1}{2}\underset{i,j}{\sum }\alpha_{i}\alpha_{j}y_{i}y_{j}K\left(x_{i},x_{j}\right),\\ \text{subject to }\overset{m}{\underset{i=1}{\sum }}y_{i}\alpha_{i}=0\text{ and }0\leq \alpha_{i}\leq C,\forall i, \end{array}$$

where *α_1_, α_2_,...,α_i _*are the Lagrangian multipliers. $K\left(x_{x},x_{j}\right)$ is the kernel function with the form: $K\left(x_{i},x_{j}\right)=\Phi \left(x_{i}\right)\cdot \Phi \left(x_{j}\right)$. The classifier based on the kernel function for a new input data ***x*_u _**is:

(10) $$g\left(x_{u}\right)=\text{sgn}\left(\overset{m}{\underset{i=1}{\sum }}y_{i}\alpha_{i}K\left(x_{i},x_{u}\right)+b\right).$$

More details about SVM can be found in [16] and [17].

In our analyses, we tested four SVM kernels, namely linear, polynomial, sigmoid and radial basis function (RBF). We optimized all the parameters, including *C, γ, r*, and degree of the kernels through an exhaustive grid search [30]. We used the 'e1071' library (v1.6-1) under the R environment to perform SVM classification.

### *k*-NN classifier

A *k*-NN classifier classifies input data according to the labels of the *k*-nearest neighbors in the training data $\text{\{}x_{i},y_{i}\text{\}}$. We calculated the Euclidean distance between a new input data point $x_{u}$, and each of the training data points $x_{i}$ using:

(11) $$d\left(x_{u},x_{i}\right)=∥x_{u}-x_{i}∥.$$

The category label of $x_{u}$ was assigned based on the majority vote of the category labels of its *k*-nearest training data (*kNN*):

(12) $$g\left(x_{u}\right)=\underset{y_{i}}{\text{argmax}}\underset{x_{i}\in kNN}{\sum }\delta \left(x_{i},y_{j}\right),$$

where $\delta \left(x_{i},y_{j}\right)\in \left\{0,1\right\}$ indicates if $x_{i}$ belongs to $y_{i}$. We tested *k *= 1, 3, 5, 7 and 9, and used the "class" library (v7.3 - 9) under the R environment to perform *k*-NN classification.

### Naive Bayes classifier

A naive Bayes classifier assumes that each of the measured features contribute independently to the probability of a category label given an input data, $p\left(y_{i}|x_{u}\right)$. According to the Bayes theorem, we have:

(13) $$p\left(y_{i}|x_{u}\right)=\frac{p\left(x_{u}|y_{i}\right)\cdot p\left(y_{i}\right)}{p\left(x_{u}\right)},$$

where $p\left(x_{u}|y_{i}\right)$ is the probability that the input data $x_{u}$ belongs to the category $y_{i}$, $p\left(y_{i}\right)$ is the probability of category $y_{i}$, and $p\left(x_{u}\right)$ is the probability of the input data $x_{u}$. The naive Bayes classifier maximizes the probability among all the possible categories:

(14) $$g\left(x_{u}\right)=\underset{y_{i}}{\text{argmax}}\left[\frac{p\left(x_{u}|y_{i}\right)\cdot p\left(y_{i}\right)}{p\left(x_{u}\right)}\right]\;\text{.}$$

As $p\left(x_{u}\right)$has the same value for a given problem, we only need to maximize the numerator of **Equation 14**. Since we assume all the features are independent, the classifier becomes:

(15) $$g^{\prime }\left(x_{u}\right)=\underset{y_{i}}{\text{argmax}}\left[p\left(y_{i}\right)\cdot \overset{}{\underset{}{\prod }}p\left(f_{i}|y_{i}\right)\right],$$

where $f_{1},f_{2},...,f_{n}$ is the set of feature values in the input data point $x_{u}$. We used the 'e1071' library (v1.6-1) under the R environment to perform naive Bayes classification.

## Results and discussion

### Random forest classification

**Figure 2:**
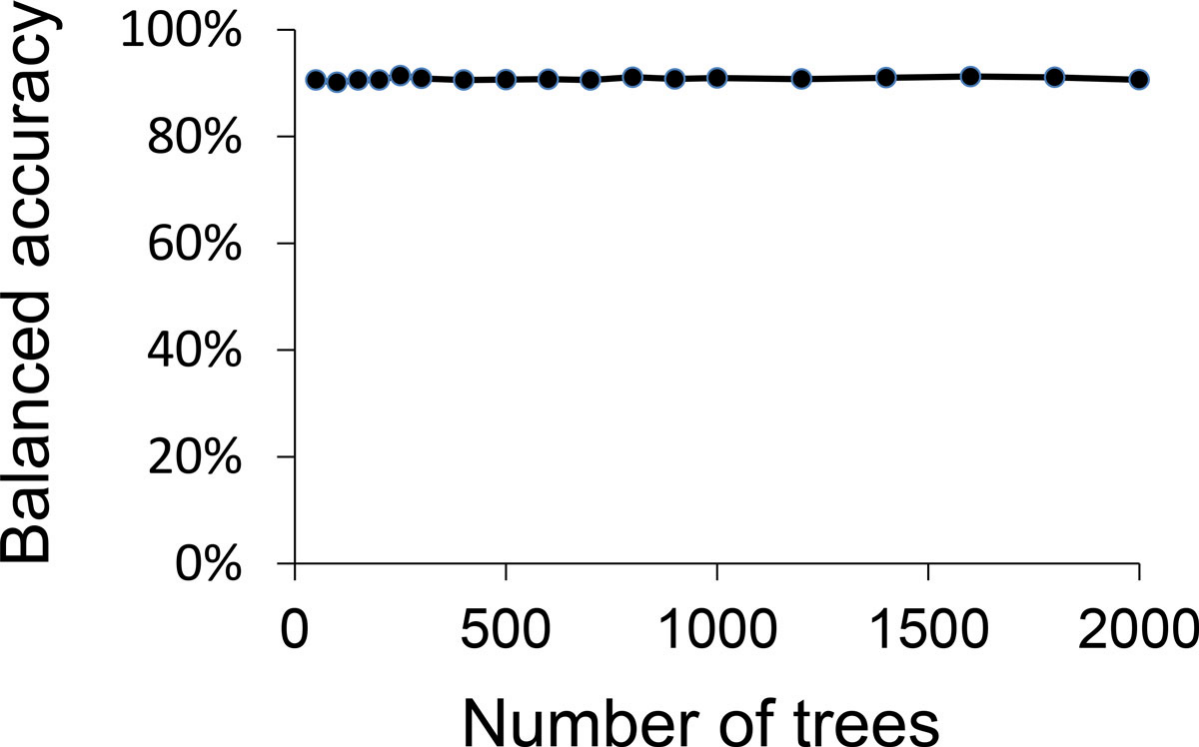
**Balanced accuracy of a random forest classifier for different numbers of trees**. The values were estimated using a 3-fold cross validation with 10 random trials on HPTC1.

We found that the performance differences between random forest classifiers using the different tested numbers of trees are very small (Figure 2). Our results agree with previous observations that random forest classifiers do not overfit even for large numbers of trees [22]. Therefore, we fixed the number of trees to be 250, which gives the highest mean balanced accuracy (87.8%), sensitivity (89.4%), and specificity (85.9%).

### SVM parameter optimization

**Figure 3:**
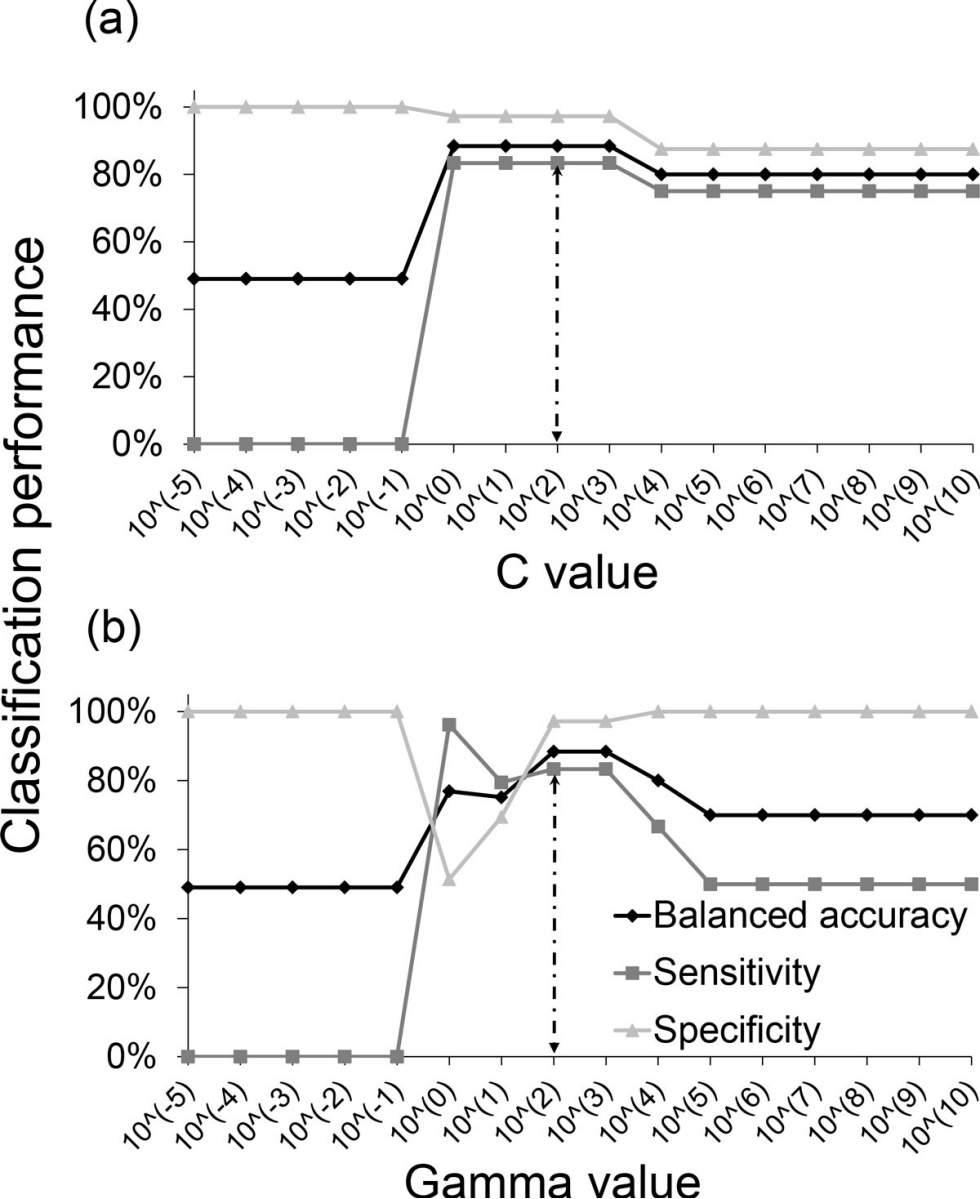
**Classification performance of a support vector machine with a radial-basis-function kernel for different parameter values**. We performed a two-dimensional grid search for the optimum values of the C and γ parameters of a SVM classifier with RBF kernel. Shown are the results for **(a) **different C values, while keeping γ = 10^2^; and **(b) **different γ values, while keeping C = 10^2^. In this example, the optimum parameters are C = 10^2 ^and γ = 10^2^.

**Figure 4:**
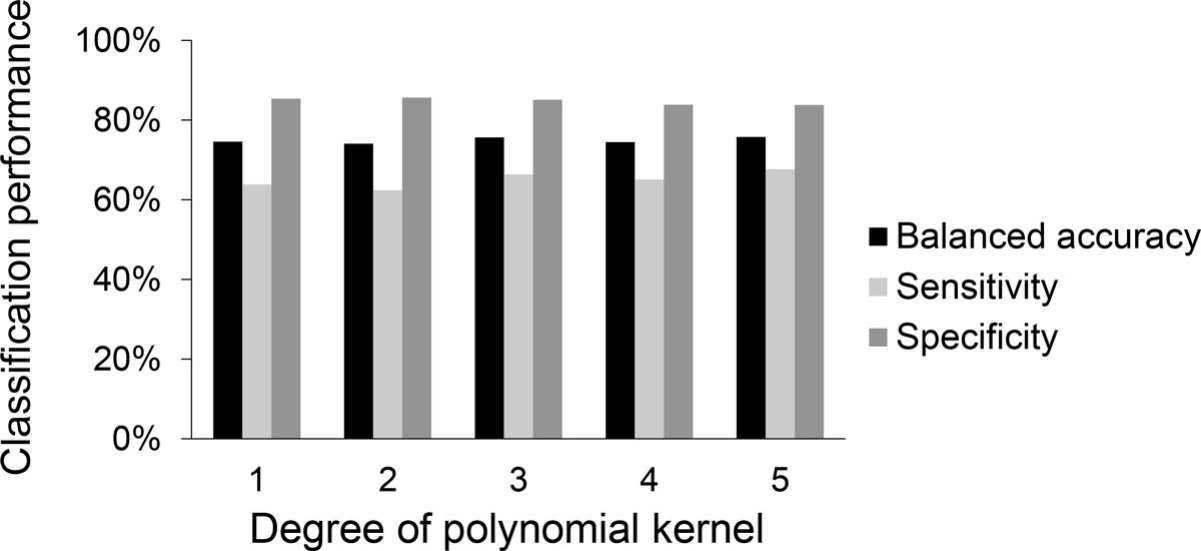
**Classification performance of a support vector machine with a polynomial kernel for different degree values**.

The classification performance of a SVM is closely related to its parameter values. A SVM classifier based on the RBF kernel has two important parameters *C *and *γ *[17]. The *C *parameter determines the misclassification penalty, and the *γ *parameter determines the width of the RBF kernel. We tested *C *and *γ *values ranging from 10^-5 ^to 10^10^. During each trial of the cross validation procedure, we always determined the optimum *C *and *γ *values based on the training data of the current fold (Figure 3). These optimum values might slightly change from fold to fold due to the different training data used. Using this optimization procedure on our 41-compound dataset, we found that the mean classification performance across all folds and trials for a RBF-based SVM classifier are 81.6% (balanced accuracy), 78.7% (sensitivity), and 84.2% (specificity). We also used similar optimization procedures to optimize the parameters of SVMs based on the linear, polynomial (Figure 4), and sigmoid kernels.

### SVM classification using linear, polynomial, sigmoid and RBF kernels

The performance of a SVM is also closely related to its kernel function. A linear kernel is simple, fast, but may not work well when the dataset is not linearly separable. Polynomial, sigmoid or RBF kernels can provide complex decision boundaries, but may also lead to the problem of overfitting [31]. To determine the best kernel for our dataset, we compared the classification performance of SVM classifiers based on linear, polynomial, sigmoid and RBF kernels using a stratified 3-fold cross validation with 10 random trials (Table 1). The parameters of these classifiers were optimized as described in the previous section. We found that the RBF kernel had the highest balanced accuracy (81.6%) and sensitivity (78.7%), and second highest specificity (84.2%). Our results suggest that the IL-6 and -8 expression levels are not linearly separable in the original feature space, and the mapping of these two features into a higher dimensional space using a RBF kernel helps to distinguish the toxic and non-toxic compounds.

**Table 1:** Classification performance of support vector machines based on different kernels.

	Linear	Polynomial	Sigmoid	RBF
**Balanced accuracy (%)**	74.6	75.8	75.7	**81.6**
**Sensitivity (%)**	63.9	67.7	70.8	**78.7**
**Specificity (%)**	**85.3**	83.8	80.7	84.2

(RBF=radial basis function. The degree of polynomial kernel is three. The values were estimated using a 3-fold cross validation procedure with 10 random trials, and averaged across three batches of HPTCs.)

### *k*-NN classification

**Figure 5:**
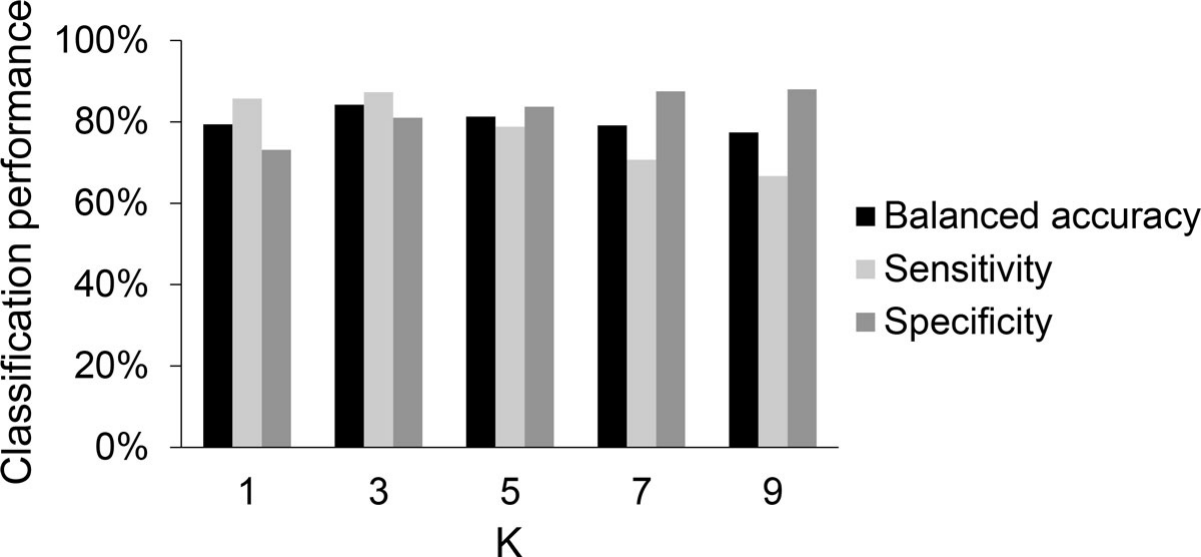
**Classification performance of a *k*-NN classifier for different numbers of nearest neighbors**. The values were estimated using a 3-fold cross validation procedure with 10 random trials on HPTC1.

We found that the optimum number of nearest neighbors (*k*) for *k*-NN classifiers is three (Figure 5). Although the mean specificity of the classifiers increases with *k*, the mean sensitivity starts to decrease after *k *= 3. At this optimum *k *value, we found that the mean classification performance across all folds and trials for *k*-NN classifiers are 74.1% (balanced accuracy), 74.0% (sensitivity), and 74.2% (specificity).

### Comparison between random forest, SVM, *k*-NN and naive Bayes classifiers

After optimizing the parameters of all the classifiers, we performed a systematic comparison of the performances of these classifiers in classifying our 41-compound dataset (Table 2). We found that random forest and SVM have the highest and second highest, respectively, values of balanced accuracy, sensitivity and specificity among all the classifiers. The *k*-NN classifier (*k *= 3) has higher sensitivity, but lower specificity, than the naive Bayes classifier. Based on these results, we conclude that a random forest classifier has the best overall performance, and will be used for all of our subsequent analyses.

**Table 2:** Classification performance of classifiers trained on both IL-6 and -8 features.

	NB	*k*-NN	SVM	RF
**Balanced accuracy (%)**	70.2	74.1	81.6	**87.8**
**Sensitivity (%)**	62.0	74.0	78.7	**89.4**
**Specificity (%)**	78.4	74.2	84.2	**85.9**

(NB=Naive Bayes classifier, ***k ***-NN=***k***-nearest neighbour classifier with ***k ***= 3, SVM=support vector machine with radial basis function kernel, RF=random forest classifier. The values were estimated using a 3-fold cross validation procedure with 10 random trials, and averaged across three batches of HPTCs.)

### Feature comparison

The expression levels of IL-6 and -8 increase in HPTCs in response to compounds that are toxic to human PTCs [14]. Previously, Li et al. found that IL-8 is more discriminative than IL-6 in classifying toxic and non-toxic compounds, but they also concluded that the combination of these two features do not provide additional advantages [14]. However, this previous analysis was performed using a classifier based on manually optimized thresholds, and the two features were thresholded independently. We wonder if multivariate classifiers, which construct decision boundaries in multi-dimensional feature spaces, may give better performance than classifiers based on individual features. Similar to the previous study, we found that random forest classifiers based on IL-8 only have higher balanced accuracy (82.8% vs. 72.8%), sensitivity (86.3% vs. 70.5%), and specificity (79.2% vs. 75.3%) than random forest classifiers based on IL-6 only (Table 3). Interestingly, we also found that the combination of IL-6 and -8 gives better classification performance than individual features (balanced accuracy = 87.8%, sensitivity = 89.4%, specificity = 85.9%). Similar trends were also observed for classifiers based on SVMs (Table 3). Our results are consistent with our findings in the previous section that IL-6 and -8 expression levels are not linearly separable, and therefore they can be better separated if a decision boundary is constructed for both features simultaneously. We conclude that both IL-8 and -6 are good and necessary features in the prediction of PTC toxicity.

**Table 3:** Classification performance of classifiers trained on different combinations of IL-6 and -8 features.

	Classifier	IL-6 only	IL-8 only	Both IL-6 and -8
**Balanced accuracy (%)**	SVM	74.1	81.4	81.6
	RF	72.8	82.8	**87.8**

**Sensitivity (%)**	SVM	66.4	78.5	78.7
	RF	70.5	86.3	**89.4**

**Specificity (%)**	SVM	81.8	84.2	84.2
	RF	75.3	79.2	**85.9**

(SVM=support vector machine, RF=random forest classifier. The values were estimated using a 3-fold cross validation procedure with 10 random trials, and averaged across three batches of HPTCs.)

### Construction of final classifiers using all compounds

**Figure 6:**
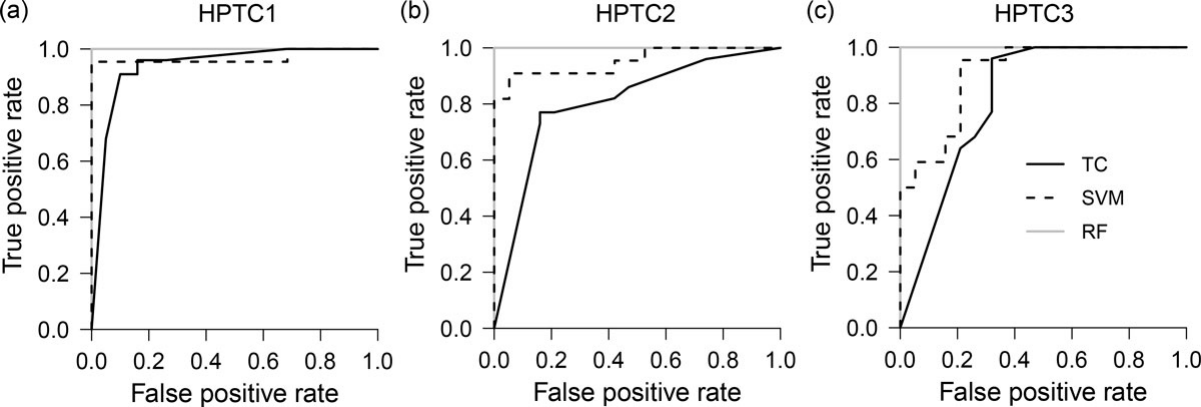
**Receiver operating characteristic curves of final classifiers trained on the whole datasets**. The ROC curves were obtained by training and testing each classifier on all compounds for three different batches of human primary renal proximal tubular cells: **(a) **HPTC1, **(b) **HPTC2, and **(c) **HPTC3 cells. (TC=threshold-based classifier from Li et al., SVM=support vector machine, RF=random forest classifier).

Finally, we trained a random forest and a SVM classifier using all the 41 compounds, and compared their classification performances to the threshold-based classifier (TC) used by Li et al. [14] (Table 4). We computed the receiver operating characteristic (ROC) curves [32] for these three classifiers (Figure 6), and measured the areas under the ROC curves (AUC). ROC curves that are closer to the upper left corner have higher AUC values and more desirable classification performances. We found that the random forest classifier has higher mean AUC (1.00 vs. 0.85), accuracy (99.3% vs. 80.7%), sensitivity (98.6% vs. 77.3%) and specificity (100.0% vs. 84.2%) than the threshold-based classifier (Table 4). The perfect AUC score indicates that the toxic and non-toxic categories can be fully separated by the random forest classifier. The SVM also performs better than the threshold-based classifier, but poorer than the random forest classifier (Table 4). We also noticed that most of the toxic compounds mis-classified by random forest classifiers are usually also mis-classified by threshold-based classifiers. For example, when using a threshold-based classifier, two compounds, namely ifosfamide and germanium oxide, were mis-classified in HPTC1 [14]; but when using our random forest classifier, only ifosfamide was mis-classified. Altogether, our results suggest that a random forest classifier based on IL-6 and -8 expression levels can be used to automatically predict drug-induced PTC toxicity.

**Table 4:** Classification performance of final classifiers trained on the whole dataset.

	Classifier	HPTC1	HPTC2	HPTC3	Mean
**AUC**	TC	0.94	0.81	0.82	0.85
	SVM	0.98	0.89	0.96	0.94
	RF	1.00	1.00	1.00	**1.00**

**Balanced accuracy (%)**	TC	90.2	80.7	71.30	80.7
	SVM	94.3	82.7	89.3	88.7
	RF	98.2	99.8	100.0	**99.3**

**Sensitivity (%)**	TC	90.9	77.3	63.6	77.3
	SVM	89.6	69.6	85.9	81.7
	RF	96.4	99.6	100.0	**98.6**

**Specificity (%)**	TC	89.5	84.2	79.0	84.2
	SVM	99.0	95.8	92.6	95.8
	RF	100.0	100.0	100.0	**100.0**

(TC=threshold-based classifier from Li et al., SVM=support vector machine, RF=random forest classifier, AUC=area under ROC curve. The values were obtained by training and test each classifier using all compounds.)

## Conclusions

In summary, we have performed a systematic comparison of the performances of four supervised classifiers, namely random forest, SVM, *k*-NN and naive Bayes classifiers, in predicting nephrotoxicity based on the IL-6 and -8 expression levels. All parameters of the classifiers were determined automatically without any user intervention. We found that random forest classifiers have the highest overall classification performance (mean balanced accuracy = 87.8%, sensitivity = 89.4%, and specificity = 85.9%). Furthermore, we also found that IL-8 is more predictive than IL-6, but a combination of both markers gives higher classification accuracy. Finally, we also show that a final random forest classifier trained automatically on the whole 41-compound dataset has higher classification accuracy than a previous threshold-based classifier [14] (mean balanced accuracy = 99.3% vs. 80.7%). This better performance is likely due to the non-linear and multivariate decision boundaries generated by the random forest classifier. Our results suggest that a random forest classifier based on these two markers can be used to automatically predict drug-induced nephrotoxicity.

Our methods are general and can be easily applied to test and identify other potential nephrotoxicity markers based on gene expression levels, metabolic profiles, or cellular phenotypes. The classification performance of our classifier may also be further increased by combining markers from these different modalities, and also by increasing the number of training compounds. An important application of our automated classifier is to predict nephrotoxicity of novel chemical compounds identified from large-scale screening of small-molecule or natural product libraries. This will allow early selection and prioritization of compound candidates for further drug development, animal tests or clinical trials, which are costly and time-consuming processes. By focusing on smaller numbers of drug candidates that are less likely to induce nephrotoxicity, the drug or compound discovery process will be more efficient, and the chance of successful clinical trials will also be increased.

## Competing interests

The authors declare that they have no competing interests.

## Authors' contributions

SR performed the computational analysis, YL and DZ provided the gene expression measurements, LH and DZ conceived of the study, and SR and LH prepared the manuscript. All authors read and approved the final manuscript.

## Supplementary Material

Additional file 1 **raw_data.csv · File format: csv file · Title of data: Raw data used in our study · Description of data: IL-6 and -8 expression levels of HPTC1, 2 and 3 treated with 41 compounds**. Click here for file

Additional file 2 **Nephrotoxicity_R_Code_v1.0.zip · File format: zip file · Title of data: Supervised classification source code · Description of data: R source code to regenerate the results in our study**. Click here for file

## Declarations

The work is supported by a grant from the Joint Council Office (JCO) Development Program, Agency for Science, Technology and Research (A*STAR), Singapore; and the Bioinformatics Institute and the Institute of Bioengineering and Nanotechnology, Biomedical Research Council, A*STAR, Singapore. Publication charges for this work was funded by the Bioinformatics Institute, Biomedical Research Council, A*STAR, Singapore.

This article has been published as part of *BMC Bioinformatics *Volume 15 Supplement 16, 2014: Thirteenth International Conference on Bioinformatics (InCoB2014): Bioinformatics. The full contents of the supplement are available online at http://www.biomedcentral.com/bmcbioinformatics/supplements/15/S16.
